# Rab7 localized on zymogen granules is involved in maturation but not in autophagy or regulated exocytosis in pancreatic acinar cells

**DOI:** 10.1038/s41598-023-49520-4

**Published:** 2023-12-12

**Authors:** Kenichi Takahashi, Hirosato Mashima, Masanari Sekine, Takeshi Uehara, Takeharu Asano, Ge-Hong Sun-Wada, Yoh Wada, Hirohide Ohnishi

**Affiliations:** 1 Department of Gastroenterology, Akita University Graduate School of Medicine, Akita, Japan; 2 Department of Gastroenterology, Jichi Medical University Saitama Medical Center, 1-847 Amanuma-Cho, Omiya-Ku, Saitama, 330-8503 Japan; 3 Department of Biochemistry, Faculty of Pharmaceutical Sciences, Doshisha Women’s College, Kyoto, Japan; 4 Division of Biological Science, Institute of Scientific and Industrial Research, Osaka University, Osaka, Japan; 5 Japan Organization of Occupational Health and Safety, Kawasaki, Kanagawa Japan

**Keywords:** Pancreas, Cell biology

## Abstract

Rab7 is known to function in the autophagy and endocytosis pathways in eukaryocytes and is related to various diseases. We recently reported that Rab7 plays a protective role against acute pancreatitis. However, its physiological function in exocytic cells remains unclear. Therefore, we investigated the role of Rab7 in pancreas-specific Rab7 knockout mice (Rab7^Δpan^). Immunofluorescence microscopy revealed that Rab7 colocalized with amylase in pancreatic acinar cells of wild-type mice, but not in Rab7^Δpan^ mice. Western blotting confirmed Rab7 localization in the zymogen granule (ZG) membranes of wild-type mice. Cholecystokinin (CCK)-stimulated amylase secretion examined using isolated pancreatic acini was similar in Rab7^Δpan^ and wild-type mice. In contrast, electron microscopy revealed that the diameters of ZGs were shorter and the number of ZGs was larger in the pancreatic acinar cells of Rab7^Δpan^ mice than in those of wild-type mice. However, the number of ZGs decreased in both Rab7^Δpan^ and wild-type mice after 24 h of starvation. In addition, the amount of amylase in the pancreas was decreased in both Rab7^Δpan^ and wild-type mice. These data indicate that Rab7 localized on ZGs plays a crucial role in the maturation of ZGs but not in their autophagy or regulated exocytosis in pancreatic acinar cells.

## Introduction

Regulated exocytosis is a highly organized cellular process that results in the secretion of various physiologically active substances, such as digestive enzymes and endocrine hormones. Pancreatic acinar cells are representative of exocytotic cells and have been utilized as a typical model in studies on regulated exocytosis and its pathways. In pancreatic acinar cells, zymogen granules (ZGs), which are secretory vesicles that pack digestive enzymes, are synthesized through the trans-Golgi network and mature during transportation toward the apical region by the intracellular vesicle transport machinery^1^. Upon stimulation of secretion through various ligand-receptor systems present at the basal membranes, ZGs dock to and fuse with the apical membranes. Digestive enzymes are then secreted into the pancreatic duct^2,3^. However, although the molecular mechanism underlying the exocytosis pathway has been intensively studied using pancreatic acinar cells as a model system, it is not fully understood.

Rab7 is a member of the Ras-related small GTP-binding protein family and is known to function in the autophagy and endocytosis pathways^4,5^. Rab7 plays a pivotal role in the maturation of autophagosomes to autolysosomes^4^. During endocytosis, Rab7 positively regulates the transition of early to late endosomes^6^ and is indispensable for lysosomal homeostasis^7^. Accordingly, Rab7 plays a crucial role in intracellular vesicle trafficking towards the lysosomes.

Recently, we reported that Rab7 in pancreatic acinar cells protects against exacerbation of acute pancreatitis^8^. In both cerulein- and L-arginine-induced experimental acute pancreatitis models, the severity of acute pancreatitis was much higher in Rab7^Δpan^ mice than in wild-type mice. In addition, intra-pancreatic trypsin activity, a major pathophysiological feature of acute pancreatitis, was extraordinarily elevated in cerulein-induced acute pancreatitis in Rab7^Δpan^ mice compared to that in wild-type mice. Since trypsinogen, trypsin pro-enzyme, is one of the major components of ZGs, we hypothesized that rab7 might play a role in the exocytosis pathway.

Therefore, in the current study, we examined the participation of rab7 in the regulated exocytosis machinery and cellular homeostasis of pancreatic acini.

## Materials and methods

### Materials

The primary antibodies used in this study were: rabbit anti-amylase (Sigma-Aldrich, St. Louis, MO, USA), rat anti-N-terminal of LAMP 1 (BD Biosciences, San Jose, CA, USA), goat anti-cathepsin B (R&D Systems, Minneapolis, MN, USA), rabbit anti-α-tubulin (Thermo Fisher Scientific, Waltham, MA, USA), and anti-Rab7 chicken monoclonal antibody, which was raised as described previously^9,10^. The secondary antibodies used in this study were: horseradish peroxidase (HRP)-conjugated donkey anti-rabbit and anti-chicken IgGs and FITC-conjugated donkey anti-rabbit and Cy3-conjugated donkey anti-chicken IgGs (Jackson ImmunoResearch, West Grove, PA, USA). SensoLyte 520 Cathepsin B and Rh110 Cathepsin L assay kits were purchased from AnaSpec (Fremont, CA, USA).

### Mice

Pancreas-specific Rab7-deficient mice were generated by crossing Rab7^flox/flox^ mice with Ptf1a-cre mice (generously gifted by Professor Yosiya Kawaguchi at Kyoto University) as described previously^8^. Littermates whose gene type was confirmed to be Rab7^flox/flox^ without the Ptf1a-Cre gene by PCR were used as wild-type mice. All experiments using mice were approved by the Institutional Animal Care and Use Committees of Akita University and Jichi Medical University. All experiments were performed in accordance with the relevant guidelines and regulations and adhered to the ARRIVE guidelines^11^ for reporting animal experiments. Mice were euthanized following ketamine/xylazine anesthesia in accordance with institutional and national guidelines for the care and use of laboratory animals and subjected to the experiments.

### Preparation of ZG membranes and western blotting

The ZG membranes were prepared as described previously^12,13^. Briefly, ZGs were isolated from the mouse pancreata using the Percall gradient centrifugation technique and lysed with nigericin. The pancreata of wild-type and Rab7^Δpan^ mice were homogenized and centrifuged at 200 × *g* for 5 min to remove cell debris. The supernatants were then centrifuged at 2000 × *g* for 15 min. The supernatants were prepared as cytosolic fractions and the resulting pellet containing mainly mitochondria and ZGs was resuspended (resuspended pellet) and subjected to Percall gradient centrifugation. After ultracentrifugation, ZG membranes were obtained as pellets. Western blotting was performed as described previously^12^, utilizing an enhanced chemiluminescence reagent to visualize the bands. The band intensity was determined using ImageJ software (National Institutes of Health, Bethesda, MD, USA).

### Immunohistochemistry and electron microscopy

Frozen sections of pancreatic tissue were used for immunohistochemical analyses. Double-staining immunofluorescence microscopy was performed as described previously^14^. Images were obtained using an LSM 780 confocal microscope (Zeiss Co., Oberkochen, Germany). Electron microscopy was performed as described previously^15^.

### Isolation of pancreatic acini and determination of amylase secretion stimulated with CCK

Pancreatic acini were isolated from the pancreata of Rab7^Δpan^ and wild-type mice by collagenase digestion as previously described^12^. Isolated acini were stimulated with CCK-8 (Peptide Institute, Osaka, Japan) at various concentrations for 30 min, as described previously^12^. Amylase released during incubation was determined using the Phadebas Amylase Test (Pharmacia Diagnostics, Columbus, OH, USA), as described previously^12^.

### Cathepsin B and L activity

To measure cathepsin B and L activity, approximately 50 mg of pancreatic tissue was homogenized using a polytron homogenizer in ice-cold assay buffer containing dithiothreitol. After centrifugation of the cell suspension, the supernatant was collected and stored at  − 80 °C until use. The assay was performed according to the manufacturer’s instructions. The activities of cathepsin B and L were normalized according to the protein concentration measured by the Bradford method, using bovine serum albumin as a standard.

### Statistical analyses

All data are shown as mean ± standard deviation. Statistical analyses were performed using analysis of variance, unless otherwise indicated. Statistical significance was set at *P* < 0.05.

## Results

### Rab7 is localized on ZG membranes in wild-type mouse pancreatic acinar cells

**Figure 1:**
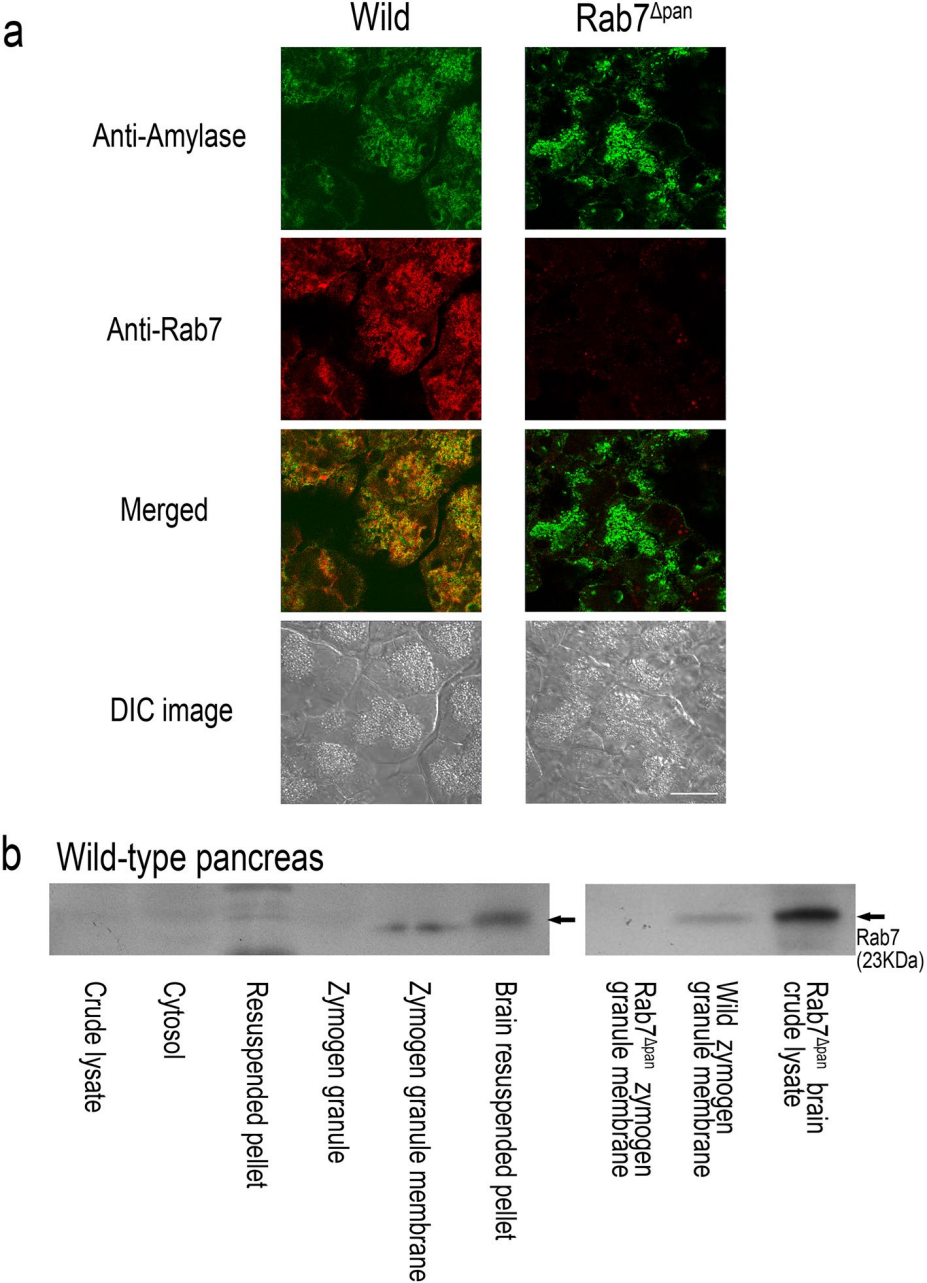
Rab7 localization on zymogen granules. (**a**) Immunofluorescence microscopy of wild-type (left column) and Rab7^Δpan^ (right column) pancreas double stained with anti-amylase (green) and anti-Rab7 (red) antibodies. Scale bar: 20 μm. (**b**) Western blotting of Rab7 using highly purified zymogen granule membranes of wild-type and Rab7^Δpan^ mice. Thirty μg of protein were loaded onto each lane. The resuspended pellet contains mainly mitochondria and zymogen granules. Brain resuspended pellet and crude lysate were used as a positive control. Original blots are presented in Supplementary Figure S1.

First, we examined the intracellular localization of Rab7 in mouse pancreatic acinar cells. As shown in Fig. 1a, immunofluorescence microscopy revealed that Rab7 colocalized with amylase in pancreatic acinar cells of wild-type mice (Fig. 1a, left column). In contrast, no discernible Rab7 signals were observed in the Rab7^Δpan^ pancreatic acinar cells (Fig. 1a, right column). These data indicate that Rab7 is localized to ZGs in mouse pancreatic acinar cells. Notably, the immunofluorescence signal of Rab7 did not completely overlap with that of amylase, implying that Rab7 is also localized to other intracellular organelles, such as late endosomes and lysosomes.

Furthermore, Western blotting of Rab7 using highly purified ZG membranes from wild-type mice confirmed the localization of Rab7 to the ZG membrane (Fig. 1b). The left panel shows that Rab7 is enriched in the ZG membrane similar to other classic ZG membrane proteins such as Rab3D^16^ and GP-2^17^. No discernible Rab7 bands were observed on the Rab7^Δpan^ ZG membrane (right panel).

### Rab7 is not involved in basal or CCK-stimulated exocytosis

**Figure 2:**
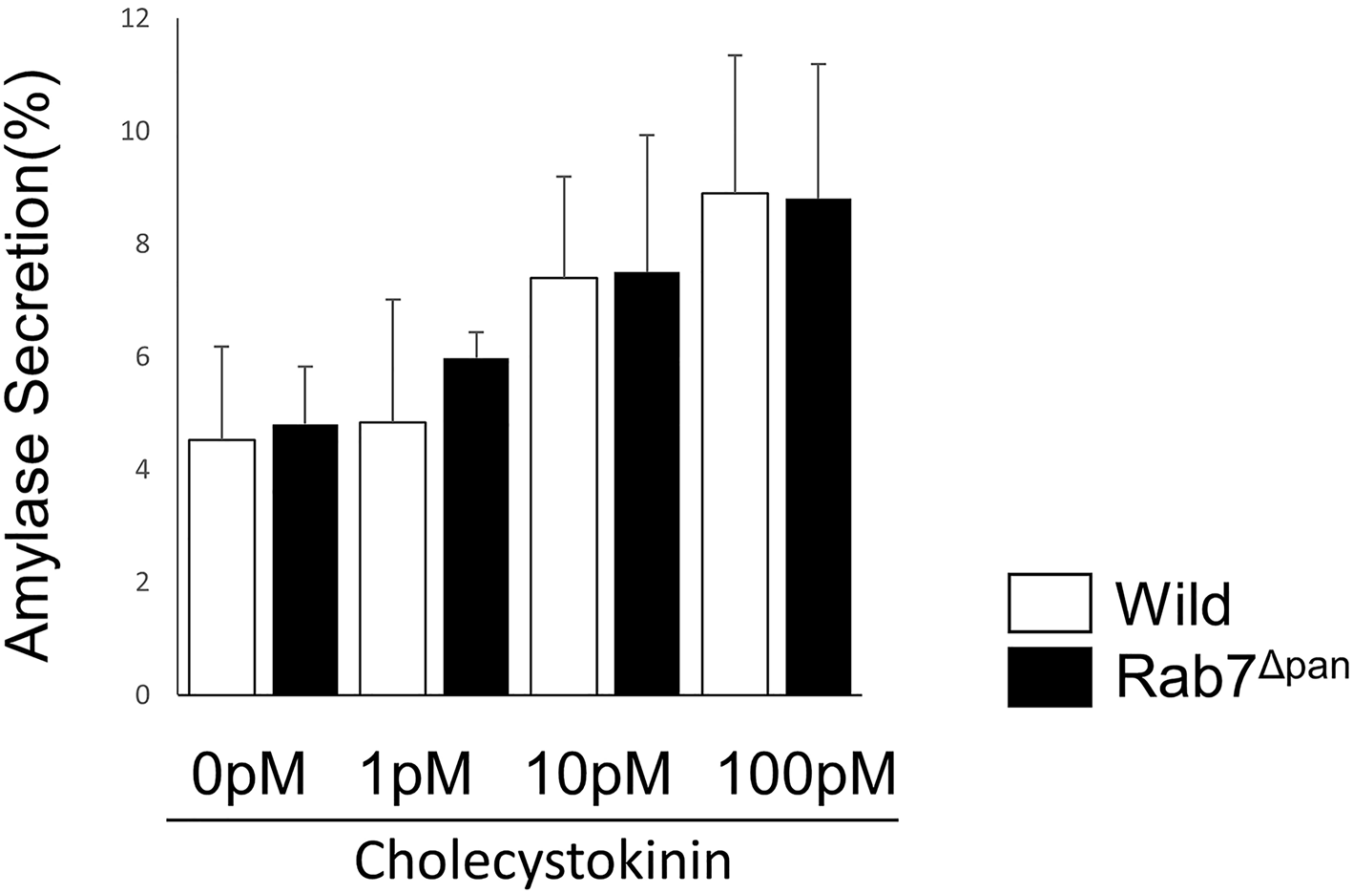
Amylase secretion from isolated pancreatic acini of wild-type (white bars) and Rab7^Δpan^ (black bars) during the incubation with the indicated concentration of CCK-8 for 30 min. Amylase secreted during the incubation was determined in triplicate and expressed as a percentage of the total amylase in the acini. The experiment was repeated three times independently with similar results.

Based on Rab7 localization to ZGs, we hypothesized that Rab7 might be involved in the regulation of exocytosis. To test this hypothesis, we compared stimulated amylase secretion using isolated pancreatic acini between wild-type and Rab7^Δpan^ mice. As shown in Fig. 2, no apparent difference was observed in either basal or stimulated amylase secretion from isolated pancreatic acini between wild-type and Rab7^Δpan^ mice. These data indicated that Rab7 localized on ZGs does not function in the regulated exocytosis of pancreatic acinar cells.

### ***The diameter of ZGs is significantly smaller and the number of ZGs is increased in Rab7***^***Δpan***^*** than in wild-type mice***

**Figure 3:**
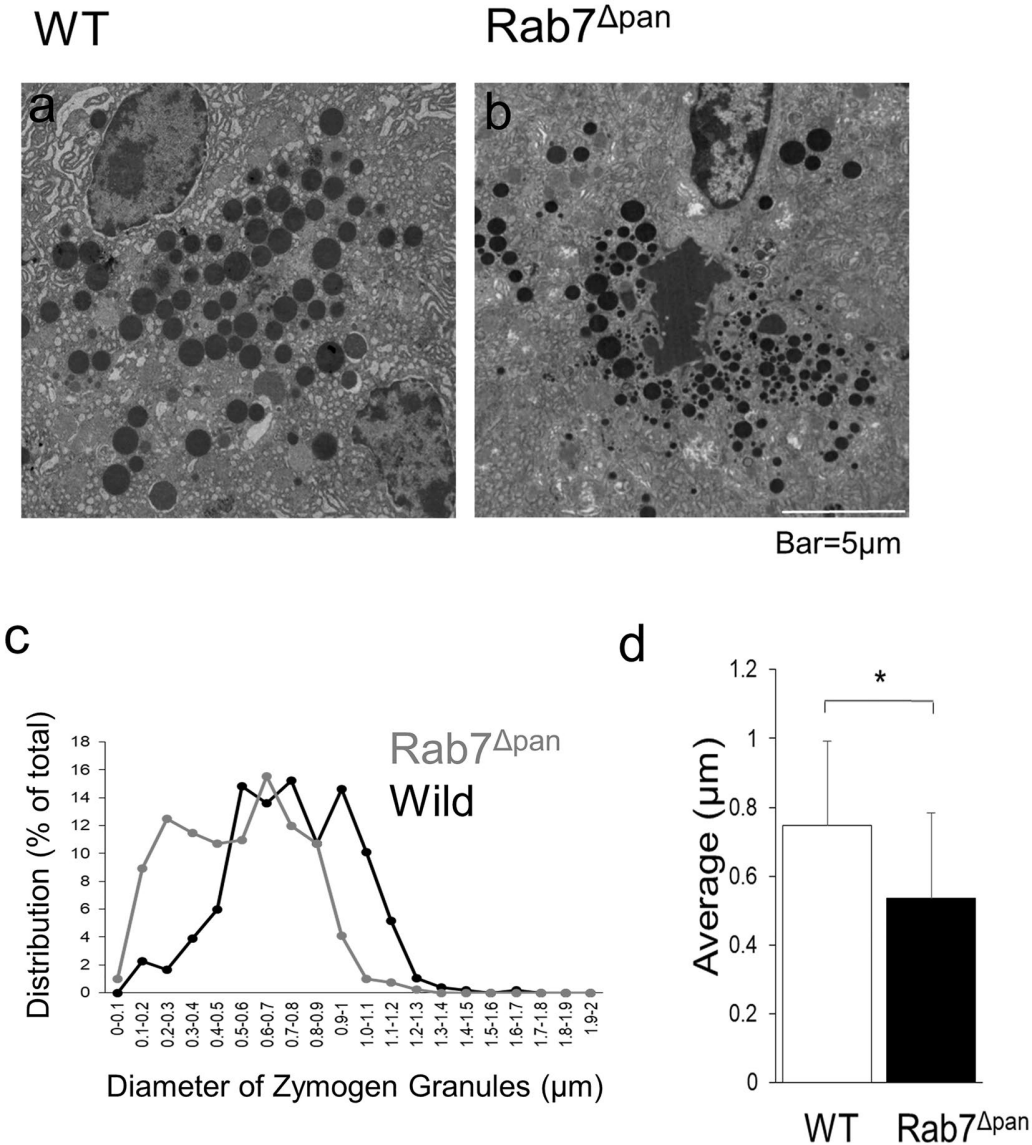
A comparison of the diameter and number of ZGs. (**a**, **b**) Electron microscopy of wild-type (**a**) and Rab7^Δpan^ (**b**) pancreas under the fed condition. Scale bar: 5 μm. Higher magnification images are shown in Supplementary Figure S2. (**c**) A comparison of the distribution of ZGs of various sizes between wild-type (black line) and Rab7^Δpan^ (gray line) pancreas. (**d**) A comparison of the average ZG diameter between wild-type (white bar) and Rab7^Δpan^ (black bar) mice. *P < 0.01 by Student’s *t*-test.

To further elucidate the function of Rab7 localized on ZGs, we examined the effect of Rab7 knockout on the size of ZGs by electron microscopy. As shown in Fig. 3, the ZGs in Rab7^Δpan^ were significantly smaller than those in wild-type mice under fed conditions (Fig. 3a–d). These data indicate that Rab7 on ZGs plays a positive role in preserving the size of the ZGs.

**Figure 4:**
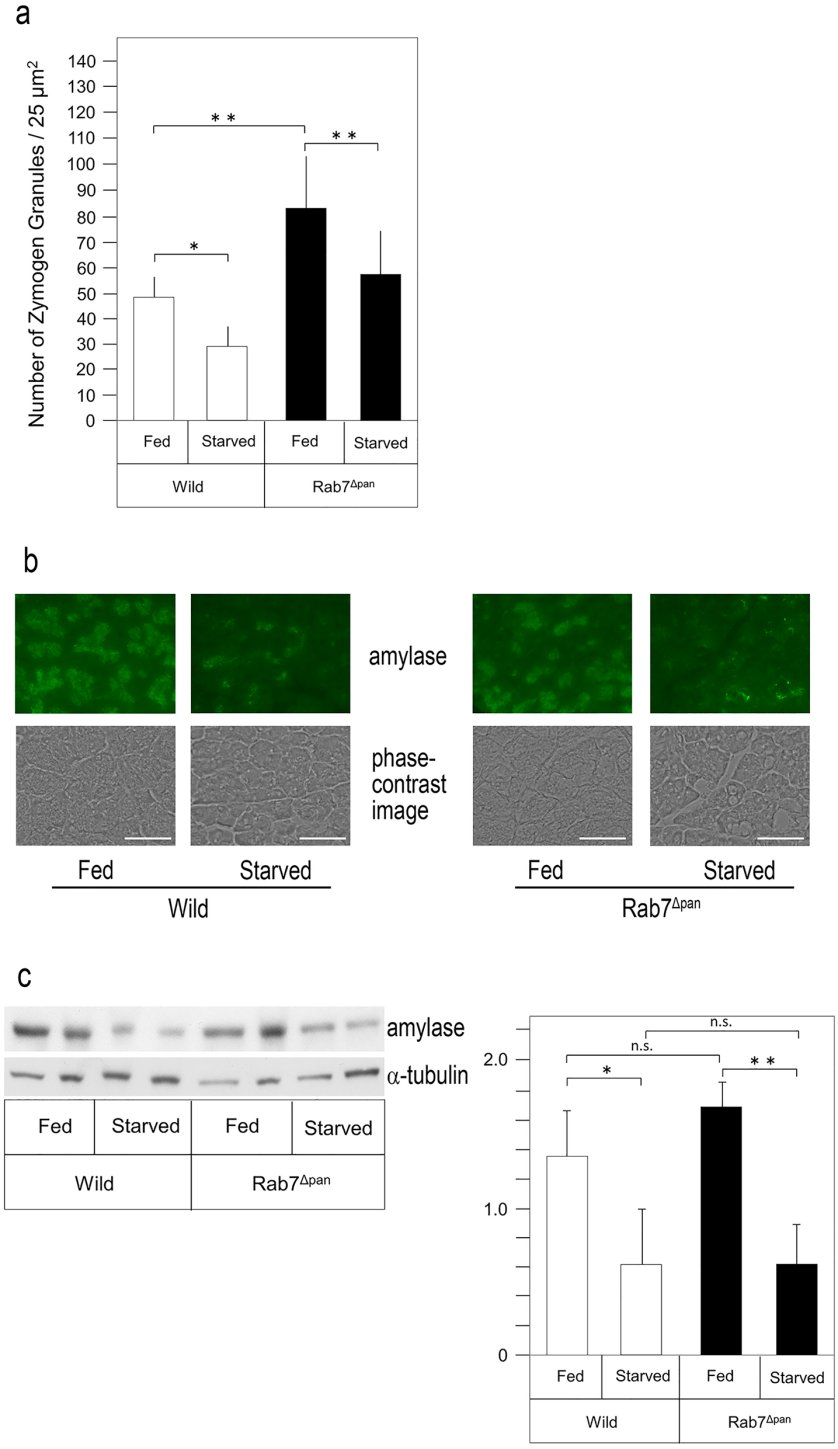
Effect of Rab7 disruption and starvation on the number of ZGs and the expression of amylase. (**a**) The number of ZGs in the pancreatic acinar cells of wild-type and Rab7^Δpan^ mice before (fed) and after (starved) 24 h of starvation were counted under electron microscopy (n = 7). *P < 0.05, **P < 0.01. (**b**) The expression of amylase in wild-type and Rab7^Δpan^ under fed and starved (24 h) conditions. The lower panel shows the phase-contrast images of the same area. Scale bar: 50 μm. (**c**) Western blotting of amylase using the pancreas lysate of wild-type and Rab7^Δpan^ mice before (fed) and after (starved) 24 h of starvation. α-tubulin was utilized as an internal control. The data are representative of two independent experiments with similar results. A densitometry analysis of the band intensity (amylase/α-tubulin, n = 4) is shown in the right panel. *P < 0.05, **P < 0.01. n.s.: not significant. Original blots are presented in Supplementary Figure S3.

Next, we investigated the effects of Rab7 disruption on the number of ZGs. As shown in Fig. 4a, under fed conditions, the number of ZGs was markedly increased (approximately 1.6-fold) in the pancreatic acinar cells of Rab7^Δpan^ mice compared with that in wild-type mice. Given that the diameter of ZGs was reduced concomitantly with an increase in their number in Rab7^Δpan^, these data strongly suggested that Rab7 promotes ZG maturation.

### Rab7 is not essential for the ZG degradation by starvation-induced autophagy

Although it has been previously reported that ZGs are degraded by starvation-induced autophagy^18^, the molecular mechanism remains uncertain. Rab7 is also known to function in autophagy^4^. Therefore, we examined whether or not Rab7 in ZGs participates in ZG degradation by starvation-induced autophagy. Therefore, we investigated the effect of starvation on the number of ZGs using electron microscopy. As shown in Fig. 4a, electron microscopic observation demonstrated that 24 h of starvation decreased the number of ZGs compared to that under fed conditions in both Rab7^Δpan^ and wild-type mice. These data indicated that Rab7 is dispensable for the degradation of ZGs by starvation-induced autophagy.

To confirm the dispensation of Rab7 in starvation-induced autophagy, we compared the amounts of amylase, a representative and major digestive enzyme packed in ZGs, in the pancreas of Rab7^Δpan^ and wild-type mice before and after starvation using immunohistochemistry and Western blotting. As shown in Fig. 4b, amylase immunoreactivity in the pancreas before starvation was comparable between Rab7^Δpan^ and wild-type mice, implying that disruption of Rab7 did not affect the amount of digestive enzymes synthesized in pancreatic acinar cells. After 24 h of starvation, immunoreactivity to amylase in the pancreas decreased in both Rab7^Δpan^ and wild-type mice compared to that under fed conditions. Western blotting confirmed these results, and starvation significantly decreased the amount of amylase in both Rab7^Δpan^ and wild-type mice (Fig. 4c). The amounts of amylase before and after starvation were comparable between the Rab7^Δpan^ and wild-type mice. These data confirmed that Rab7 is not essential for the degradation of ZGs by starvation-induced autophagy.

### ***Cathepsin B is activated by the degradation of LAMP1 in Rab7***^***Δpan***^

In our previous study, lysosomal functions were affected in Rab7^Δpan^, and the autophagy flux was impaired between autophagosome and autolysosome processes during starvation^8^. Lysosomal enzymes play a key role in the degradation of ZGs via starvation-induced autophagy^18^. However, starvation-induced degradation of ZGs was not altered in Rab7^Δpan^. Therefore, we compared the lysosomal function under fed and starved conditions.

**Figure 5:**
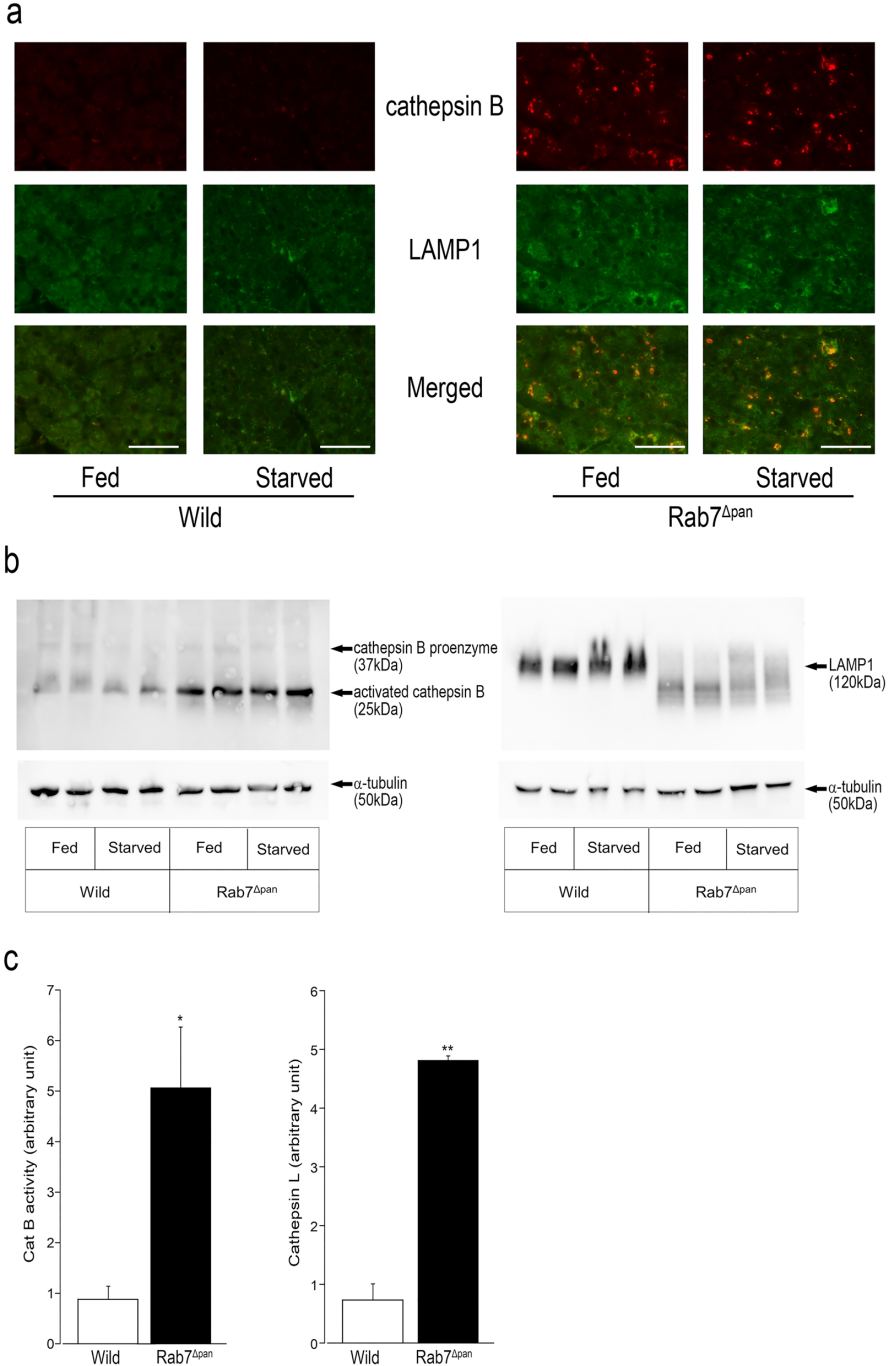
A comparison of the expression of cathepsin and LAMP1 in wild-type and Rab7^Δpan^ under fed and starved conditions (24 h). (**a**) Immunofluorescence microscopy. Scale bar: 50 μm. (**b**) Western blotting: α-tubulin was used as an internal control. Data are representative of two independent experiments with similar results. Original blots are presented in Supplementary Figure S4 and S5. (**c**) A fluorometric analysis of cathepsin B and L activity under physiological feeding conditions. The experiment (n = 3 for each group) was independently repeated twice, with similar results. *P < 0.05, **P < 0.01 by Student’s *t*-test.

Lysosomal functions, including the expression of cathepsin B, a representative lysosomal protease, and LAMP-1, which is essential for lysosomal functions^19^ under fed conditions, were evaluated in our previous study^8^. To determine the effect of Rab7 disruption under starved conditions on these lysosomal functions and to compare the changes between both conditions, we evaluated lysosomal functions under fed and starved conditions simultaneously. As shown in Fig. 5a, cathepsin B expression in Rab7^Δpan^ increased under physiological feeding conditions and was comparable to that observed under starved conditions. Likewise, Western blotting (Fig. 5b) showed that the expression of the double-chain form (a mature active form) of Cathepsin B was much elevated in Rab7^Δpan^ pancreas under both fed and starved conditions. Immunohistochemistry (Fig. 5a) showed that the immunoreactivity of LAMP-1 was fairly increased in Rab7^Δpan^ compared to the wild type under both fed and starved conditions. Expression was stronger around the perinuclear region, suggesting that lysosomal localization was not altered in Rab7^Δpan^. Western blotting showed that the band of LAMP-1 shifted to a lower molecular position with laddering in Rab7^Δpan^ under both fed and starved conditions, suggesting the degradation of LAMP-1 in Rab7^Δpan^. The extent of degradation appeared to be stronger under the starved conditions than under the fed conditions.

Cathepsin B converts trypsinogen into trypsin, whereas cathepsin L degrades both trypsinogen and trypsin. To confirm the overexpression of cathepsin in Rab7^Δpan^, we evaluated the activity of cathepsins B and L under basal feeding conditions using a fluorometric assay kit. As shown in Fig. 5c, the activities of cathepsin B and L were significantly elevated in Rab7^Δpan^ mice compared to wild-type mice.

## Discussion

In the current study, immunofluorescence microscopy and Western blotting demonstrated the localization of Rab7 in the ZGs of pancreatic acinar cells. Although both basal and stimulated amylase secretions from isolated pancreatic acini were comparable between wild-type and Rab7^Δpan^ mice, electron microscopic analysis revealed that the sizes of ZGs were significantly smaller, and reciprocally, the number of ZGs was much larger in Rab7^Δpan^ than in wild-type mice. However, the machinery of starvation-induced degradation of ZGs was suggested to be maintained in the pancreatic acini of Rab7^Δpan^ mice. These data indicate that Rab7 localized on ZGs in pancreatic acinar cells plays a role in the regulated exocytosis machinery to preserve ZGs at a precise size and number, but not in digestive enzyme secretion or ZG degradation by autophagy during starvation. Rab7 plays an important role in lysosomal homeostasis.

Rab7 functions have been intensively studied with respect to endocytosis and autophagy pathways, both of which are pathways toward lysosomes. In the endocytosis pathway, Rab7 plays a role in selective cargo pathway sorting of early endosomes^20^ and in the intracellular trafficking of late endosomes^21,22^. Rab7 is required for its normal progression^4^. Consistent with this knowledge, we recently reported that Rab7 is involved and positively functions in both endocytosis and autophagy pathways in pancreatic acinar cells^8^. In the present study, we expanded our previous work by elucidating a novel function of Rab7 in the exocytosis pathway. Localized on ZGs, Rab7 plays an important role in maintaining its precise size and number. To our knowledge, this is the first report to demonstrate that Rab7 is localized on secretory vesicles and is involved in the regulation of the exocytosis machinery.

Secretory vesicles in exocytotic cells, including pancreatic acinar ZGs, are synthesized in the trans-Golgi network and mature during the transition towards the apical membrane^23,24^. The maturation steps consist of the fusion of immature small secretory granules, leading to the enlargement of vesicles to the precise size of the matured secretory vesicles^23^. In the pancreatic acinar cells of Rab7^Δpan^, shrinkage in size and an increase in the number of ZGs were observed. Therefore, it is reasonable to conclude that Rab7 plays a role in the maturation of ZGs. It is widely accepted that mammalian Rab7 regulates the autophagosome-lysosome fusion step^4,5^. Kuchitsu et al. reported that Rab7 is involved in autolysosome maturation under nutrient-rich conditions using Rab7 knockout mammalian cultured cells ^25,26^. Regarding the localization of ZGs, ZGs were localized around the apical region of Rab7^Δpan^ pancreatic acinar cells (Fig. 3a), suggesting that Rab7 did not play a role in the transportation of ZGs toward the apical membrane.

Currently, multiple members of the Rab protein family, including Rab3D^16^, Rab4^14^, Rab27A^27^ and 27B^28^, are known to localize to ZGs and play various roles in pancreatic acinar exocytosis. Gene disruption techniques have been frequently utilized in functional analyses of Rab proteins on ZGs in pancreatic acinar cells. Hou et al.^29^ reported that Rab27B is localized on ZGs and that its gene disruption in mice results in small-sized ZGs, which is consistent with our current study. However, inhibition of stimulated amylase secretion from isolated pancreatic acini has also been observed in Rab27B knockout mice^29^. These reports imply that Rab7 and Rab27B exert similar positive effects on ZG maturation but also indicate that the functions of these two Rab proteins are distinct at the digestive enzyme secretion step. Interestingly, Riedel et al.^30^ previously reported that Rab3D knockout mice exhibited markedly enlarged ZGs in pancreatic acinar cells compared to wild-type mice without alteration of stimulated digestive enzyme secretion. Taken together, the exocytosis machinery of pancreatic acinar cells, especially during ZG maturation, is substantially systematized and preserved by multiple Rab proteins on the ZGs.

ZGs pack various digestive enzymes, including proteinases, which are essential for food digestion and nutrient absorption. Proteinases are retained in ZGs in their inactive form. However, once proteinases are activated in pancreatic acinar cells due to pancreatic damage and/or excessive accumulation of ZGs in pancreatic acinar cells, they induce autolysis, leading to acute pancreatitis^31^. Therefore, it has been postulated that the excessive accumulation of ZGs in pancreatic acinar cells is decreased through an intracellular vesicle traffic mechanism^32,33^ to prevent autolysis of pancreatic acinar cells. Mizushima et al.^18^ clearly demonstrated using transgenic mice expressing green fluorescent protein-tagged LC3 that ZGs retained in pancreatic acinar cells due to starvation were sequestered in autophagosomes and degraded. Furthermore, Rab7 has been demonstrated to play essential roles in autophagy pathways^4,26^. In addition, we recently reported that the autophagy pathway in Rab7^Δpan^ pancreatic acinar cells deteriorates during autophagosome maturation^8^. Thus, we hypothesized that rab7, localized on ZGs, participates in ZG degradation by autophagy during starvation.

However, our present experiments using electron microscopy, immunohistochemistry, and Western blotting indicated that Rab7 disruption did not affect ZG degradation via starvation-induced autophagy. Regarding the mechanisms involved, the following is speculated: (1) although the autophagy pathway is deteriorated in Rab7^Δpan^ pancreatic acinar cells, they might still retain the capacity to degrade ZGs, which is a high-risk factor for their own homeostasis under starvation condition^31^, or (2) another autophagy pathway specific to ZG degradation, such as zymophagy^34^, independent of Rab7, might degrade ZGs in Rab7^Δpan^ pancreatic acinar cells.

A recent report stated that the syntaxin7-SNAP29-YKT6 complex mediates autophagosome-lysosome fusion, even in the absence of Rab7^26,35^. In Rab7^Δpan^ acinar cells, LAMP-1 was suspected to be degraded, and the activation of cathepsin B and L was observed even under fed conditions. These activated proteases may be ready for degradation of unnecessary ZGs under starvation conditions. Transient expression of dominant negative EGFP-Rab7T22N causes the dispersal of lysosomes into the cytosol of HeLa cells^7^. However, the immunoreactivity of LAMP-1 accumulated in the perinuclear region of Rab7^Δpan^ pancreatic acinar cells, and its localization was not altered (Fig. 5a). Thus, there may be an alternative signal for lysosomes that compensates for the absence of Rab7. Further studies are required to elucidate the molecular mechanisms underlying ZG autophagy during starvation.

In conclusion, we showed that Rab7 is localized to the ZGs of pancreatic acinar cells and plays a pivotal role in their maturation. This knowledge provides novel insights that will further our understanding of the molecular mechanisms underlying the regulated exocytosis machinery.

### Supplementary Information

Supplementary Figures.

## Data Availability

The datasets generated and/or analyzed during the current study are available from the corresponding author upon reasonable request.
