# Supplementary material for: Rab7 localized on zymogen granules is involved in maturation but not in autophagy or regulated exocytosis in pancreatic acinar cells
Source: Sci Rep. 2023 Dec 12;13:22084. doi: 10.1038/s41598-023-49520-4 (PMC10716180; doi:10.1038/s41598-023-49520-4)

Supplementary Figure S1
(Full Blots for Figure 1b)

Rab7

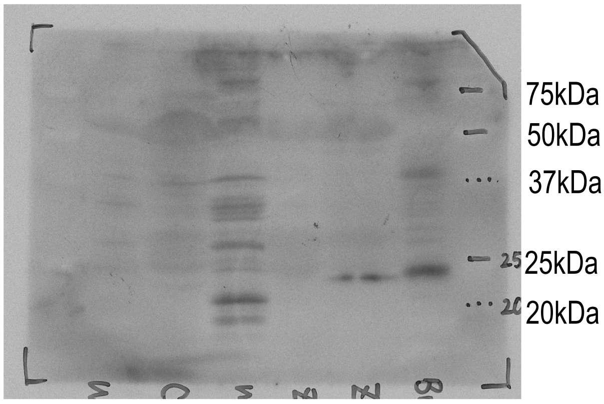

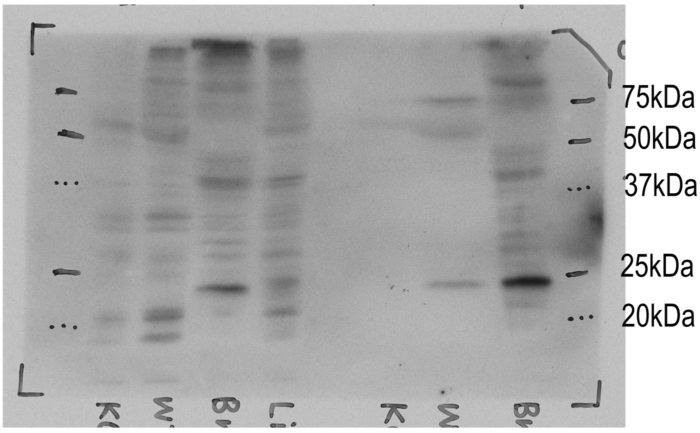

Supplementary Figure S2
(High magnification images of Figure 3a, b)

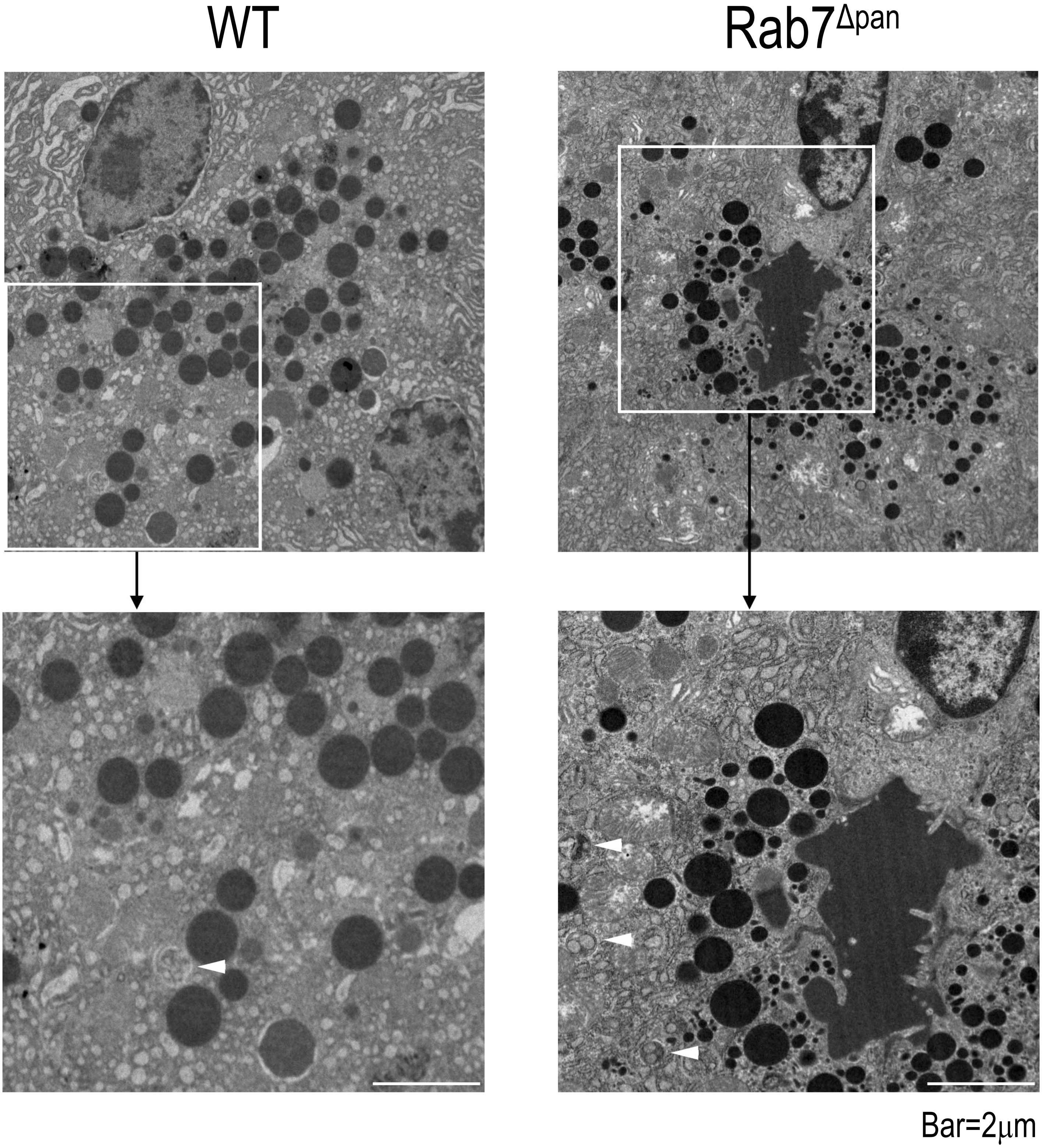

Arrowheads indicate autophagic vacuoles.
Autophagic vacuoles were frequently observed in the acinar cells of $Rab7^{\Delta pan}$ even in the fed condition, but were rarely observed in those of wild-type mice.

Supplementary Figure S3
(Full Blots for Figure 4c)

amylase

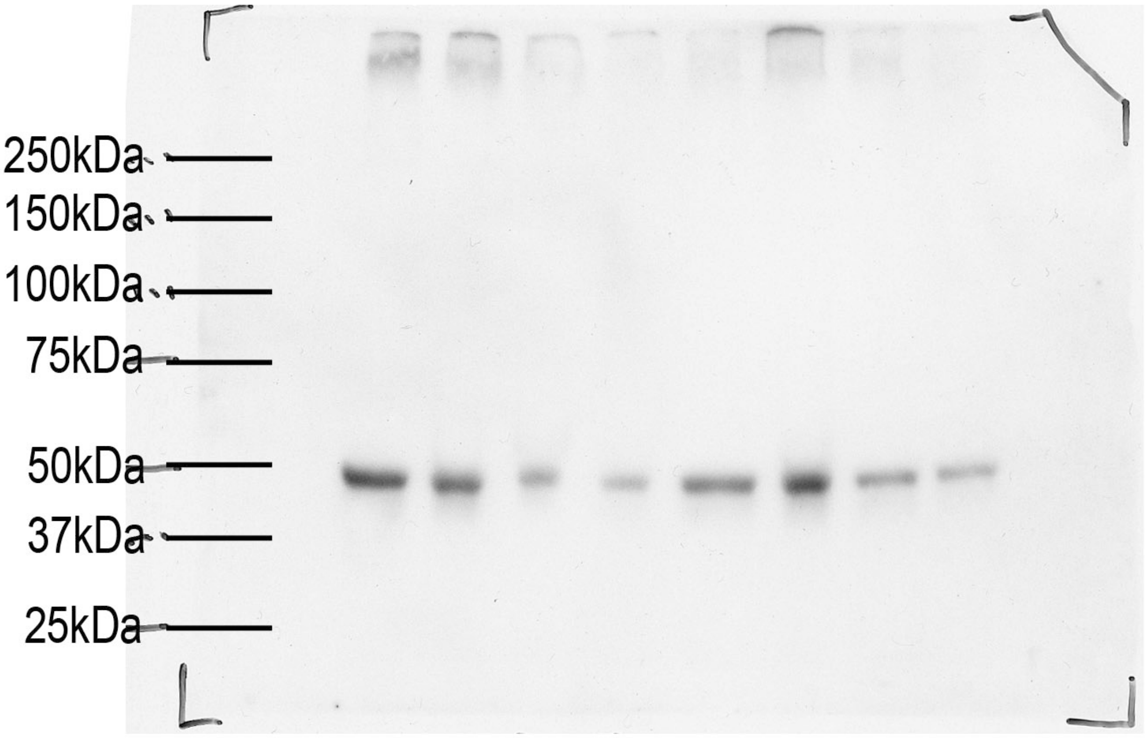

α-tubulin

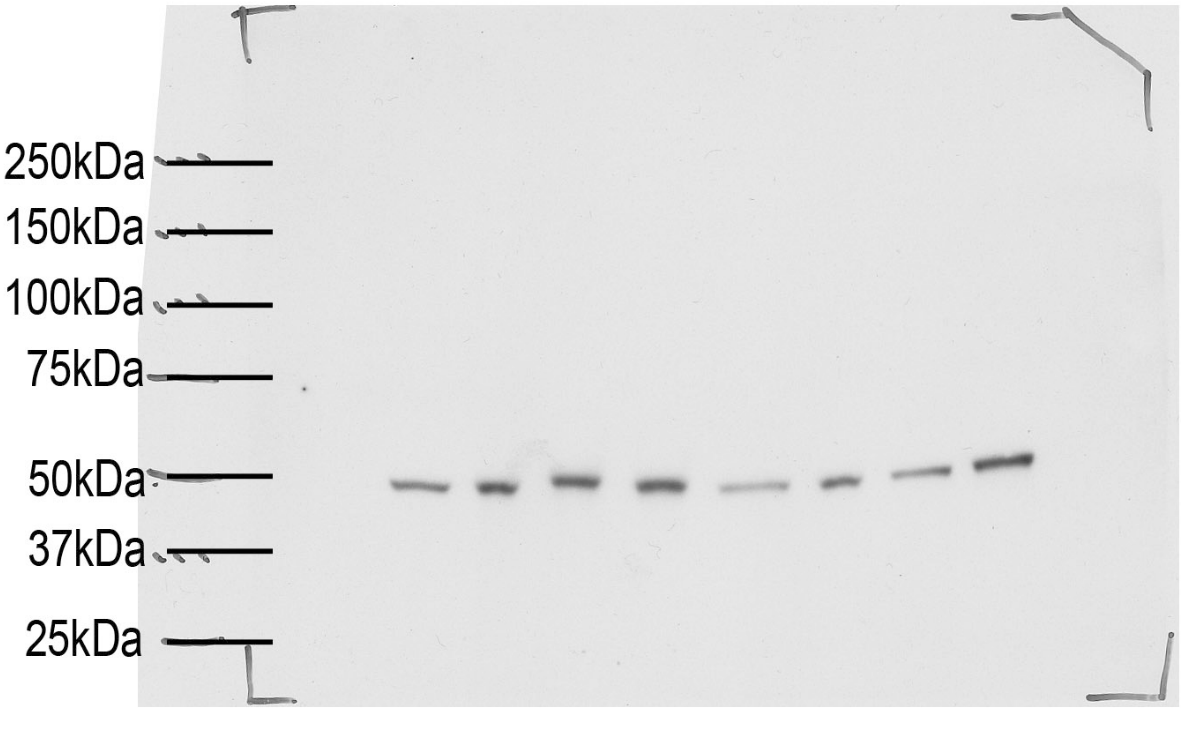

Supplementary Figure S4
(Full Blots for Figure 5b left panel)

cathepsin B

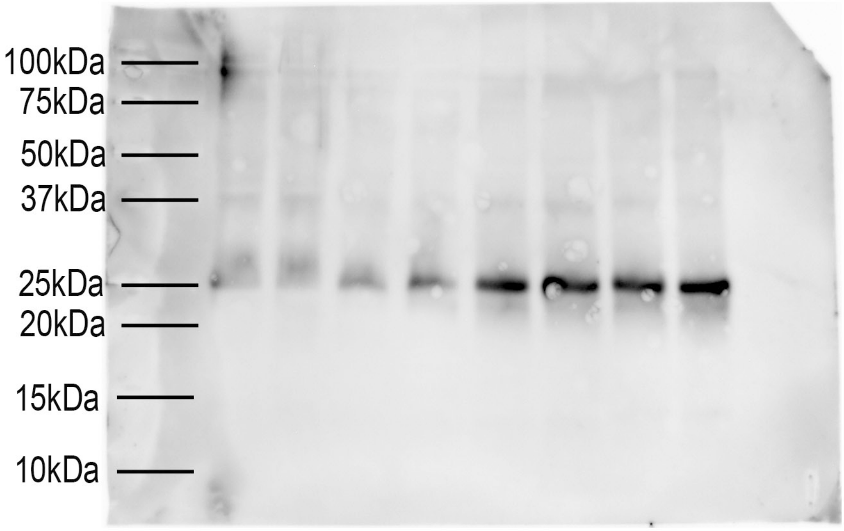

α-tubulin

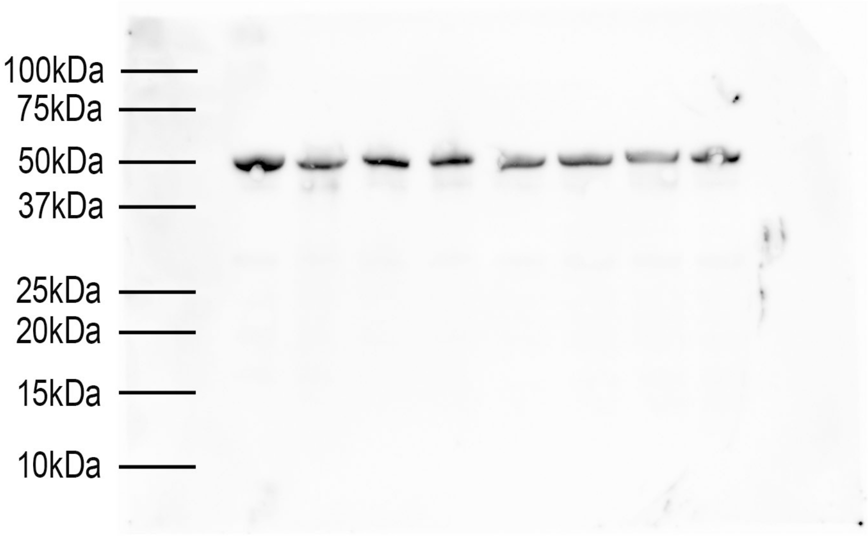

Supplementary Figure S5
(Full Blots for Figure 5b right panel)

LAMP1

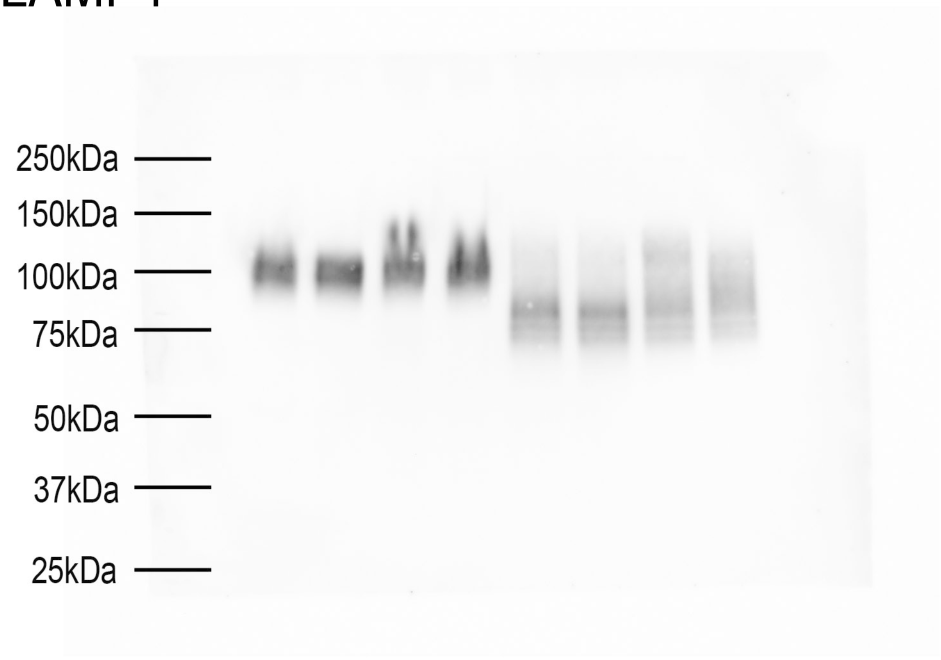

α-tubulin

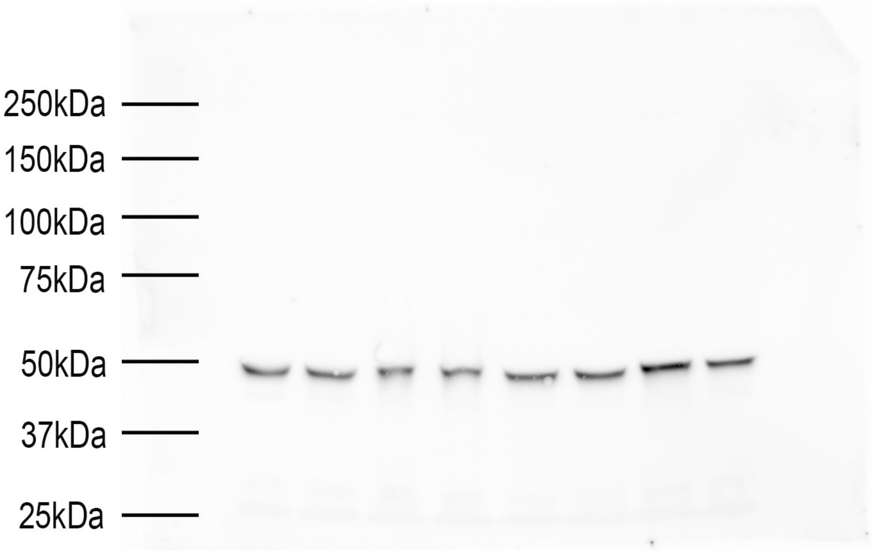

Supplement: Supplementary file 1 — Supplementary Figures. [file 41598_2023_49520_MOESM1_ESM.pdf]
